# Structural and biochemical analyses of the nuclear pore complex component ELYS identify residues responsible for nucleosome binding

**DOI:** 10.1038/s42003-019-0385-7

**Published:** 2019-05-03

**Authors:** Wataru Kobayashi, Yoshimasa Takizawa, Maya Aihara, Lumi Negishi, Hajime Ishii, Hitoshi Kurumizaka

**Affiliations:** 1 Laboratory of Chromatin Structure and Function, Institute for Quantitative Biosciences, The University of Tokyo, 1-1-1 Yayoi, Bunkyo-ku, Tokyo, 113-0032 Japan; 2 Graduate School of Advanced Science and Engineering, Waseda University, 2-2 Wakamatsu-cho, Shinjuku-ku, Tokyo, 162-8480 Japan

**Keywords:** Nucleosomes, Cryoelectron microscopy

## Abstract

The nuclear pore complex embedded within the nuclear envelope is the essential architecture for trafficking macromolecules, such as proteins and RNAs, between the cytoplasm and nucleus. The nuclear pore complex assembly occurs on chromatin in the post-mitotic phase of the cell cycle. ELYS (MEL-28/AHCTF1) binds to the nucleosome, which is the basic chromatin unit, and promotes assembly of the complex around the chromosomes in cells. Here we show that the Arg-Arg-Lys (RRK) stretch of the C-terminal ELYS region plays an essential role in the nucleosome binding. The cryo-EM structure and the crosslinking mass spectrometry reveal that the ELYS C-terminal region directly binds to the acidic patch of the nucleosome. These results provide mechanistic insight into the ELYS-nucleosome interaction, which promotes the post-mitotic nuclear pore complex formation around chromosomes in cells.

## Abstract (web summary)

Wataru Kobayashi et al. use cryo-EM to study the C-terminal ELYS subunit of the nuclear pore complex. They show that a three-residue stretch binds to the acidic patch of the nucleosome, providing new mechanistic insights into ELYS nucleosome interactions.

## Introduction

In eukaryotes, genomic DNA is organized into chromosomes and is accommodated within the nucleus. Histones are the major protein components of chromosomes and form the nucleosome as a basic unit^1^. In the nucleosome, 145–147 base pairs of DNA segments are lefthandedly wrapped 1.65 turns around the histone octamer, composed of two each of histones H2A, H2B, H3, and H4^2–4^. The nucleosomes are connected by short linker DNA segments and appear as chromatin with a beads-on-a-string configuration^1^.

The chromosome conformation dynamically changes during the cell cycle^5,6^. In interphase cells, the chromosomes are diffused within the nucleus, which is encircled by the nuclear envelope^5,6^. The nuclear pore complexes (NPCs) are embedded in the nuclear envelope, and materials, such as proteins and RNAs, are transported between the nucleus and cytoplasm through the NPCs^7–9^. In higher eukaryotes, the NPCs are disassembled concomitant with the breakdown of the nuclear envelope in mitotic cells. The duplicated chromosomes are then segregated into the dividing cells^10–12^. After the chromosome segregation, reassembly of the nuclear envelope and NPCs occurs at segregated chromosomes, and then the functional nucleus is established in the daughter cells^10,11,13–15^.

A nucleoporin, ELYS, plays a key role in post-mitotic NPC assembly^16–18^. ELYS localizes to the nuclear rim in interphase cells and recruits NPC components, such as the Nup107-160 sub-complex, to chromosomes^16–18^. The ELYS-knockdown HeLa cells are severely defective in the NPC formation^16–18^. Consistently, in *Caenorhabditis elegans*, the knockdown of the ELYS homologue, MEL-28, induces an NPC assembly deficiency^19,20^. These findings suggested that ELYS functions as a primer for the post-mitotic NPC reassembly on chromosomes^11,13,15–18,21^.

In agreement with the idea that ELYS mediates the NPC assembly on chromosomes, ELYS reportedly binds to chromatin with both the AT-hook DNA-binding domain^18,22^ and C-terminal region^23^. ELYS directly binds to the histone H2A-H2B dimer and the nucleosome in vitro^23^. Intriguingly, histone depletion in mouse zygotes or *Xenopus* egg extracts resulted in defective NPC assembly^23,24^. These results suggest that the chromatin binding of ELYS is essential for the NPC assembly. However, the mechanistic details of the ELYS–nucleosome interaction have not been elucidated.

In the present study, we purified the ELYS C-terminal peptide containing the AT-hook DNA-binding domain and a conserved Arg-Arg-Lys (RRK) stretch. Biochemical analyses revealed that the ELYS C-terminal peptide efficiently binds to the nucleosome, and its RRK stretch, in addition to the AT-hook DNA-binding domain, may play an essential role for the nucleosome binding. Cryo-electron microscopy (cryo-EM) and crosslinking mass spectrometric analyses revealed that the ELYS C-terminal peptide binds to the acidic patches of the nucleosome surface. These presented data clarify how ELYS binds to the nucleosome.

## Results

### The RRK stretch of ELYS is important for nucleosome binding

**Fig. 1:**
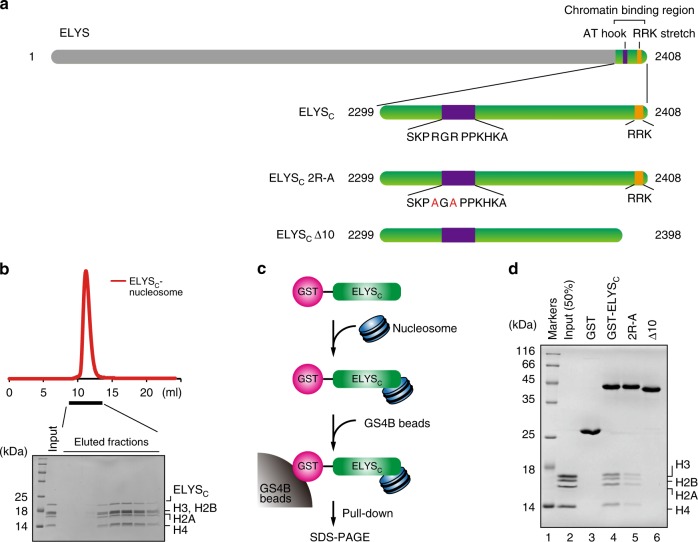
Nucleosome binding by ELYS_C_. **a** The *Xenopus laevis* ELYS_C_ fragments used in this study. The purple and orange boxes represent the regions corresponding to the AT-hook DNA-binding domain and the RRK stretch, respectively. In the ELYS_C_ 2R-A mutant, the Arg2332 and Arg2334 residues in the AT-hook DNA binding domain are replaced by Ala. The amino acid residues are numbered. In the ELYS_C_ Δ10 mutant, the C-terminal ten residues including the RRK stretch are deleted. The amino acid residues are numbered. **b** Gel-filtration analysis of the ELYS_C_–nucleosome complex. ELYS_C_ and the nucleosome containing the 145 bp Widom 601 DNA were mixed in a 2.5:1 molar ratio, at room temperature for 20 min. The red line indicates the elution profile of the ELYS_C_–nucleosome complex. Eluted fractions were analyzed by 18% sodium dodecyl sulfate-polyacrylamide gel electrophoresis (SDS-PAGE) with Coomassie Brilliant Blue staining. The uncropped gel image is shown in Supplementary Fig. 4. **c** Experimental scheme for the pull-down assay with GS4B beads. **d** The pull-down assay for the ELYS_C_–nucleosome interaction. Lane 1 represents molecular mass markers. Lane 2 indicates input nucleosome (50%). Lane 3 indicates a negative control experiment with glutathione *S*-transferase (GST). Lanes 4–6 indicate the experiments with GST-ELYS_C_, GST-ELYS_C_ 2R-A, and GST-ELYS_C_ Δ10. The samples were analyzed by 18% SDS-PAGE with Coomassie Brilliant Blue staining. Experiments were independently repeated four times, and consistent results were obtained. The uncropped gel image is shown in Supplementary Fig. 4

The *Xenopus laevis* ELYS protein is composed of 2408 amino acid residues, and its C-terminal region is reportedly involved in chromatin binding^18,22–26^. The ELYS peptide containing amino acid residues 2281–2408 has been reported as a nucleosome binding fragment^22,23^. We first purified the ELYS 2281–2408 peptide and found that the ELYS 2299–2408 fragment was somewhat resistant to trypsin proteolysis. We then prepared the C-terminal ELYS peptide containing amino acid residues 2299–2408 (named ELYS_C_) (Fig. 1a and Supplementary Fig. 1a). The nucleosome was reconstituted with a 145 base-pair (bp) Widom 601 sequence, which does not appear in linker DNA regions, thus eliminating possible interactions between ELYS_C_ and linker DNAs (Supplementary Fig. 1b). A gel filtration analysis revealed that ELYS_C_ stoichiometrically bound to the nucleosome (Fig. 1b). We then prepared ELYS_C_ as a glutathione *S*-transferase (GST)-fusion protein (Supplementary Fig. 1c) and performed the pull-down assay (Fig. 1c). ELYS_C_ contains the AT-hook DNA-binding domain, which reportedly binds to chromatin^18,22,23,26^. The RRK stretch, composed of the Arg2404, Arg2405, and Lys2406 residues, exists near the C-terminal end (Fig. 1a). Consistent with the previous reports^23,24^, the ELYS_C_ peptide efficiently bound to the nucleosome, and the AT-hook mutant (ELYS_C_ 2R-A), in which the ELYS Arg2332 and Arg2334 residues are replaced by Ala, drastically reduced its nucleosome binding, probably due to defective DNA binding (Fig. 1d, lanes 4 and 5). Interestingly, we found that the deletion of the C-terminal ten amino acid residues of ELYS_C_ (ELYS_C_ Δ10) abolished its nucleosome-binding ability (Fig. 1d, lane 6). This ELYS_C_ deletion mutant lacked the RRK stretch but retained the AT-hook domain, suggesting that the AT-hook residues may not bind histones. Therefore, the RRK stretch may primarily function in the nucleosome binding.

### The ELYS basic residues are required for nucleosome binding

**Fig. 2:**
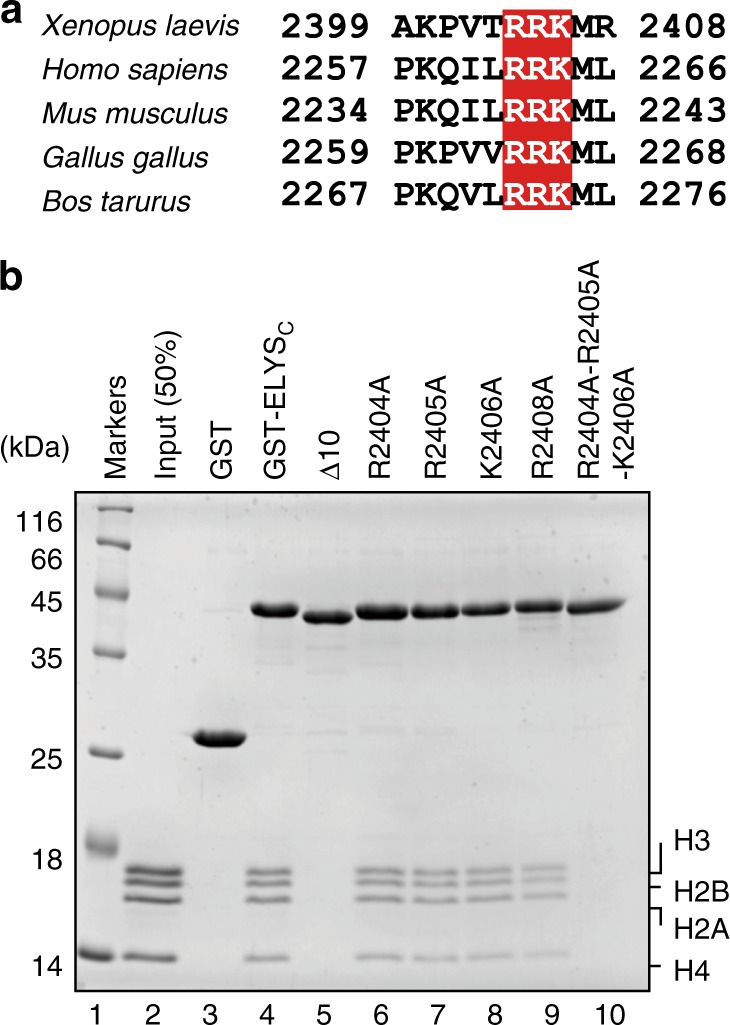
The RRK stretch at the C-terminus of ELYS is important for nucleosome binding. **a** Sequence alignment of the *Xenopus laevis*, *Homo sapiens*, *Mus musculus*, *Gallus gallus*, and *Bos taurus* ELYS proteins. The RRK stretch is colored red. The amino acid residues are numbered. **b** The pull-down assay for the nucleosome with GST, GST-ELYS_C_, or GST-ELYS_C_ mutants. Lane 1 represents molecular mass markers. Lane 2 indicates input nucleosome (50%). Lane 3 indicates a negative control experiment with GST. Lanes 4–10 represent the experiments with GST-ELYS_C_ and GST-ELYS_C_ mutants (R2404A, R2405A, K2406A, R2408A, and R2404A-R2405A-K2406A), respectively. Experiments were independently repeated three times, and consistent results were obtained. The uncropped gel image is shown in Supplementary Fig. 4

To identify the ELYS_C_ amino acid residues responsible for the nucleosome binding, we performed a mutational analysis. The basic amino acid residues, such as Arg and Lys, potentially bind to the DNA backbone phosphates or the acidic patch of the nucleosome surface^27^. The C-terminal 10 amino acid residues of ELYS include four basic amino acid residues, Arg2404, Arg2405, Lys2406, and Arg2408 (Fig. 2a). To determine whether these basic amino acid residues are involved in the nucleosome binding, we prepared the ELYS_C_ mutants, ELYS_C_(R2404A), ELYS_C_(R2405A), ELYS_C_(K2406A), and ELYS_C_(R2408A), in which the ELYS Arg2404, Arg2405, Lys2406, and Arg2408 residues were each replaced by Ala (Supplementary Fig. 1c). Interestingly, the amino acid substitution at each basic residue slightly decreased the nucleosome binding, but the effect was not substantial (Fig. 2b, lanes 6–9). In contrast, the nucleosome binding was almost abolished when the Arg2404, Arg2405, and Lys2406 residues of the RRK stretch were simultaneously replaced by Ala (Fig. 2b, lane 10). These results suggested that the RRK stretch is responsible for the nucleosome binding of ELYS.

### The cryo-EM structure of the ELYS_C_–nucleosome complex

**Fig. 3:**
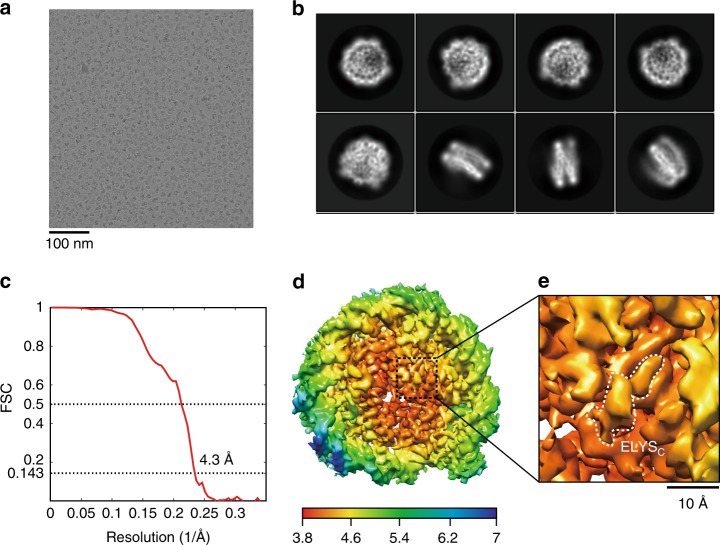
The cryo-electron microscopic structure of the ELYS_C_–nucleosome complex. **a** Digital micrograph of the ELYS_C_–nucleosome complexes. Scale bar indicates 100 nm. **b** Selected two-dimensional class averages from single particle images of the ELYS_C_–nucleosome complex. The box size is 21 nm^2^. **c** Fourier shell correlation (FSC) curve after gold-standard map refinement. The overall resolution of the ELYS_C_–nucleosome complex is 4.3 Å at an FSC = 0.143. **d** Local resolution map of the ELYS_C_–nucleosome complex, showing the resolution range across the map from 3.8 to 7 Å. **e** Enlarged view of the ELYS density with a scale bar

**Fig. 4:**
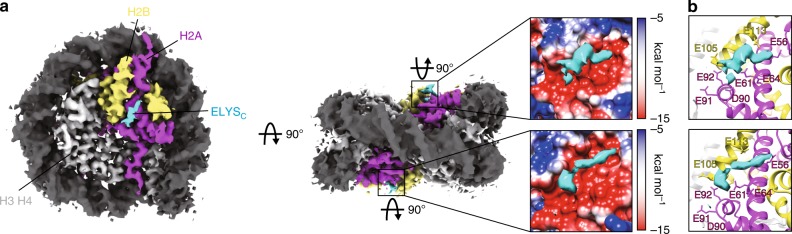
ELYS_C_ interacts with the acidic patch of the nucleosome. **a** Cryo-electron microscopic structure of the ELYS_C_–nucleosome complexes at 4.3 Å, contoured at 3.7 sigma above mean density. ELYS_C_, H2A, and H2B are colored cyan, magenta, and yellow, respectively. Enlarged views around the acidic patches encircled with rectangles are presented. The acidic (red) and basic (blue) regions of the nucleosome surface are colored according to the Coulombic surface charge. The extra density corresponding to part of ELYS_C_ is colored cyan. **b** The acidic amino acid residues of the nucleosome surface around the ELYS_C_ region are presented. The H2A Glu56, Glu61, Glu64, Asp90, Glu91, and Glu92 residues are colored magenta. The H2B Glu105 and Glu113 residues are colored yellow

We performed a cryo-EM analysis of the ELYS_C_–nucleosome complex. The nucleosome reconstituted with a 145 base-pair Widom 601 sequence was incubated with the GST-fused ELYS_C_ peptide, and the ELYS_C_–nucleosome complex was fixed with paraformaldehyde and purified by the GraFix method. The cryo-EM images of the ELYS_C_–nucleosome complexes were collected (Fig. 3a). The cryo-EM structure of the ELYS_C_–nucleosome complex was reconstructed and refined to 4.3 Å resolution (Fig. 3b–d, Supplementary Fig. 2, and Table 1). In the structure, extra densities were clearly visible on the acidic patches of both surfaces of the nucleosome (Fig. 4a, b). These extra densities observed on the acidic patch may represent part of ELYS_C_ bound to the nucleosome. Although the shapes of these extra densities are slightly different, their density sizes are similar. Therefore, we suspected that these extra densities may be responsible for the same ELYS_C_ region.

**Table 1:** Cryo-EM data collection and image processing

Data collection and processing	ELYS_C_–nucleosome (EMDB-9802)
Magnification	×100,000
Voltage (kV)	200
Electron exposure (e–/Å^2^)	~56
Defocus range (μm)	−1.5 to −3.0
Pixel size (Å)	1.32
Symmetry imposed	C1
Initial particle images (no.)	1,300,821
Final particle images (no.)	81,008
Map resolution (Å)	4.3
FSC threshold	0.143
Accuracy of rotations (˚)	1.904
Accuracy of translations (pix)	0.618
Map sharpening B factor (Å^2^)	−218

*Cryo-EM* cryo-electron microscopy, *FSC* Fourier shell correlation

### Crosslinking mass spectrometric analysis

**Fig. 5:**
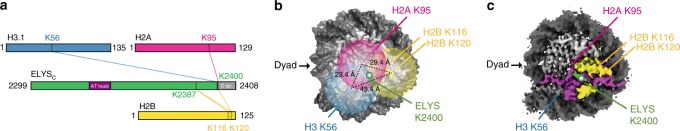
The ELYS–nucleosome interaction determined by crosslinking mass spectrometry. **a** Schematic representation of the crosslinking mass spectrometric analysis. The interlinks between histone core regions (H2A, H2B, and H3.1) and ELYS_C_ are depicted with lines. The amino acid residues involved in the interlinks are indicated with numbers. The purple and gray boxes represent the regions corresponding to the AT-hook DNA-binding domain and the C-terminal ten amino acid residues, respectively. H3.1, H2A, H2B, and ELYS_C_ are colored blue, magenta, yellow, and green, respectively. **b** Possible location of the ELYS Lys2400 residue, predicted by the crosslinking mass spectrometric analysis. The black dashed lines with numbers show the distances between the Cα atoms of the H2A Lys95, H2B Lys120, and H3 Lys56 residues. The red circle represents the 20 Å radius, which indicates the possible crosslinking area of the H2A Lys95 residue by DSS-H12/D12 (the central point is the Cα atom). The yellow circle represents the 20 Å radius, which indicates the possible crosslinking area of the H2B Lys120 residue by DSS-H12/D12 (the central point is the Cα atom). The blue circle represents the 20 Å radius, which indicates the possible crosslinking area of the H3.1 Lys56 residue by DSS-H12/D12 (the central point is the Cα atom). The possible location of the ELYS Lys2400 residue is indicated as a green sphere. The crystal structure of the nucleosome containing a 145 -bp Widom 601 DNA (PDB ID: 3lZ0) was used^39^. The black arrow indicates the dyad axis of the nucleosome. **c** The cryo-electron microscopic structure of the ELYS_C_–nucleosome complex. The predicted location of ELYS Lys2400 is superimposed on the structure

We performed a crosslinking mass spectrometric analysis. In this experiment, we used the crosslinker DSS-H12/D12, which mediates crosslinking between Lys residues. We found that the ELYS Lys2400 residue, which is located near the RRK stretch, crosslinked with the H2A Lys95, H2B Lys120, and H3 Lys56 residues (Fig. 5a and Supplementary Fig. 3). These results indicated that the distances between the ELYS Lys2400 residue and the H2A Lys95, H2B Lys120, and H3 Lys56 residues are within 10–20 Å, because the crosslinking distance of DSS-H12/D12 is 10–20 Å^28^. The ELYS Lys2406 residue in the RRK stretch was not detected as a crosslinked residue, probably due to its tight binding to the acidic residues in the acidic patch of the nucleosome. We then mapped the possible crosslinking areas for the H2A Lys95, H2B Lys120, and H3 Lys56 residues on the nucleosome structure, as colored circles with a 20-Å radius (Fig. 5b). The location of the ELYS Lys2400 residue was predicted in the area overlapped by these three circles (Fig. 5b). Interestingly, the predicted ELYS Lys2400 location overlaps with the extra density corresponding to part of ELYS_C_ in the cryo-EM structure (Fig. 5c). The ELYS Lys2400 residue exists near the ELYS RRK stretch (from 2404 to 2406), and the H2A Lys95, H2B Lys116, and H2B Lys120 residues are located near the acidic patch of the nucleosome. Therefore, the ELYS_C_ RRK stretch may directly bind to the acidic patch of the nucleosome.

The Lys residue that tightly bound to the acidic amino acid residue may not be crosslinked by DSS-H12/D12, because of steric hindrance. Therefore, the ELYS Lys2400 residue may be efficiently crosslinked with the Lys residues in the histone, because it may not tightly bind to the histone residues, although its localization must be very close to the RRK stretch.

## Discussion

ELYS plays a key role in the post-mitotic NPC assembly mediated by nucleosomes^16–18^. In fact, ELYS reportedly binds to the nucleosome in vitro and co-purified with histones in vivo^23,24^. However, the mechanism of the ELYS–nucleosome binding has not yet been elucidated. In the present study, we reconstructed the structure of the ELYS_C_–nucleosome complex by the cryo-EM method (Figs. 3 and 4). The cryo-EM structure, combined with the crosslinking mass spectrometry, demonstrated that the ELYS C-terminal region including the RRK stretch is important for binding to the acidic patch on the nucleosome surface (Figs. 3–5). Consistently, biochemical analyses revealed that the Arg and Lys residues in the ELYS RRK stretch function in the nucleosome binding (Figs. 1 and 2).

The RRK stretch containing Arg-Arg-Lys residues is highly conserved among ELYS orthologs (Fig. 2a). However, the functional importance of these residues has not been clarified. Here we found that the RRK stretch functions in binding to the nucleosomal acidic patch. The acidic patch comprises the H2A and H2B acidic amino acid residues, which are exposed on both sides of the nucleosome surface (Fig. 4). The acidic patches of the nucleosome provide binding sites for chromatin-associated factors, such as RCC1, Sir3, PRC1, CENP-C, and LANA^29–33^. In these proteins, the arginine side chain specifically binds to the acidic patch on the nucleosome and is referred to as the arginine anchor^27^. In the ELYS_C_–nucleosome complex, the arginine residues in the RRK stretch of ELYS_C_ may also function as an arginine anchor. Our mutational analyses showed that all three basic residues, but not each residue, in the RRK stretch are required for the nucleosome binding (Fig. 2). Therefore, the binding mechanism of ELYS_C_ to the acidic patch of the nucleosome may be somewhat different from that of the conventional arginine anchor motif. Alternatively, one of the two arginine residues in the RRK stretch may redundantly function as an arginine anchor.

Consistent with previous results, our biochemical analyses revealed that the AT-hook region also functions in nucleosome binding (Fig. 1). Importantly, our mutational analyses demonstrated that the contribution of the RRK stretch may be higher than that of the AT-hook region in nucleosome binding (Fig. 1). In the ELYS_C_–nucleosome complex structure, the AT-hook region was not visible, because of its flexible nature. The AT-hook region of ELYS reportedly binds to DNA^22,23^. The AT-hook motifs of other proteins reportedly prefer to bind to DNA-containing AT-rich regions^22^. Therefore, the DNA binding of the AT-hook region may support the ELYS nucleosome binding, which is mainly mediated by the binding of the RRK stretch to the acidic patch.

In vitro analyses revealed that importin β and transportin directly bind to the C-terminal region of ELYS (2359–2408) but not the AT-hook region^34^. The importin β and transportin-binding region of ELYS contains the RRK stretch. Therefore, ELYS–nucleosome binding may compete with importin β and transportin binding and may inhibit ELYS recruitment to chromatin^34,35^. Thus the release of ELYS from importin β and transportin may be a prerequisite for NPC assembly^34,35^. RanGTP reportedly weakens the ELYS–importin β and ELYS–transportin interactions^34,35^. Therefore, the RanGTP-mediated ELYS release from importin β and transportin may be important for the ELYS–chromatin association, which is the primary step for NPC assembly in the post-mitotic phase.

## Methods

### Purification of ELYS fragments and histones

Human histones H2A, H2B, H3.1, and H4 were produced in *Escherichia coli* cells^36,37^. The denatured hexahistidine (His_6_)-tagged human histone proteins were purified by chromatography using nickel-nitrilotriacetic acid (Ni-NTA) agarose beads (Qiagen). The His_6_-tag portion was removed by thrombin protease treatment. After removal of the His_6_-tag portion, the samples were further purified by chromatography using MonoS column (GE Healthcare). For the reconstitution of the histone octamer^36,37^, four core histones (H2A, H2B, H3.1, and H4) were equally mixed in denaturing buffer (20 mM Tris-HCl (pH 7.5), 1 mM EDTA, 7 M guanidine hydrochloride, and 20 mM 2-mercaptoethanol). The reconstituted histone octamer was further purified by chromatography using Superdex 200 gel-filtration column (GE Healthcare).

The DNA fragment encoding *X. laevis* ELYS 2299–2408 (ELYS_C_) was synthesized by the polymerase chain reaction (PCR) method. The amplified DNA fragment encoding ELYS_C_ was ligated into the *Nde*I site, which was artificially created by PCR mutagenesis in the pGEX-6p-1 vector. The GST-tagged ELYS_C_ protein was expressed in the *E. coli* BL21 (DE3) codon plus RIL strain (Stratagene). The cells were cultured at 30 °C, and the expression of GST-ELYS_C_ was induced when the cell growth reached an optical density of 0.6 (OD_600_), by adding 0.5 mM IPTG and further culturing at 18 °C. The cells producing GST-ELYS_C_ were collected and resuspended in resuspension buffer 1, containing 50 mM Tris-HCl (pH 7.5), 500 mM NaCl, 1 mM EDTA, 10% glycerol, and 2 mM 2-mercaptoethanol. The cells were disrupted by sonication, and the supernatant was separated from the cell debris by centrifugation. The supernatant was gently mixed with Glutathione Sepharose 4B beads (GE Healthcare) at 4 °C for 1 h. The beads were packed into an Econo-column (Bio-Rad) and were washed with 50 column volumes of resuspension buffer 1. GST-ELYS_C_ was eluted by 30 column volumes of 50 mM Tris-HCl (pH 8.8) buffer, containing 500 mM NaCl, 1 mM EDTA, 20 mM reduced glutathione, and 2 mM 2-mercaptoethanol. The eluted sample was immediately applied to a HiLoad 16/60 Superdex 200 gel filtration column (GE Healthcare) equilibrated with 50 mM HEPES-NaOH (pH 7.5) buffer, containing 150 mM NaCl and 2 mM 2-mercaptoethanol. Aliquots of purified GST-ELYS_C_ were frozen in liquid nitrogen and stored at −80 °C. The GST-ELYS_C_ mutants (2R-A, Δ10, R2404A, R2405A, K2406A, R2408A, and R2404A-R2405A-K2406A) were generated using a KOD-plus-mutagenesis Kit (TOYOBO), and the resulting ELYS_C_ mutants were purified by the same method as the wild-type ELYS_C_ purification.

For the purification of His_6_-tagged ELYS_C_, the N-terminally His_6_-tagged ELYS_C_ protein was produced in the *E. coli* BL21 (DE3) codon plus RIL strain. The cells were cultured at 30 °C to an OD_600_ = 0.6, and His_6_-ELYS_C_ expression was induced by adding 0.2 mM IPTG and incubating the culture further at 18 °C. The cells producing His_6_-ELYS_C_ were resuspended in resuspension buffer 2, containing 50 mM Tris-HCl (pH 8.0), 500 mM NaCl, 5 mM imidazole, 10% glycerol, and 2 mM 2-mercaptoethanol. The cell debris was removed by centrifugation. The supernatant was gently mixed with Ni-NTA agarose beads (Qiagen) at 4 °C for 1 h, and then the Ni-NTA beads were washed with 50 column volumes of resuspension buffer 2. The proteins bound to the Ni-NTA beads were eluted by a linear gradient of 5–500 mM imidazole. The fractions containing His_6_-ELYS_C_ were collected, and the His_6_-tag portion was removed by thrombin protease treatment (2 units/mg, GE Healthcare) during dialysis against 20 mM Tris-HCl (pH 7.5) buffer, containing 200 mM NaCl, 1 mM EDTA, 10% glycerol, and 2 mM 2-mercaptoethanol. After the His_6_-tag portion was removed, the sample was loaded onto a MonoS column (GE Healthcare). The column was washed with 10 column volumes of 50 mM Tris-HCl (pH 8.0) buffer, containing 200 mM NaCl, 1 mM EDTA, 10% glycerol, and 2 mM 2-mercaptoethanol. The protein was eluted by a 30 column volume linear gradient of 200-900 mM NaCl. The peak fraction containing ELYS_C_ was immediately applied to a HiLoad 26/60 Superdex 75 gel filtration column (GE Healthcare) equilibrated with 20 mM HEPES-NaOH (pH 7.5) buffer, containing 300 mM NaCl and 1 mM dithiothreitol (DTT). Aliquots of purified ELYS_C_ were frozen in liquid nitrogen and stored at −80 °C.

The concentrations of the ELYS_C_ and GST-tagged ELYS_C_ proteins were determined by sodium dodecyl sulfate-polyacrylamide gel electrophoresis (SDS-PAGE), with bovine serum albumin (BSA) as the standard protein.

### Purification of nucleosomes

For the nucleosome reconstitution, the 145-bp DNA fragment containing the Widom 601 DNA was prepared^38,39^. The nucleosomes were reconstituted by the salt dialysis method and prepared by polyacrylamide gel (6%) electrophoresis, using a Prep Cell apparatus (Bio-Rad). The nucleosomes were concentrated with an Amicon Ultra centrifugal filter unit (Millipore).

### Gel-filtration analysis

ELYS_C_ and the nucleosome containing the 145-bp Widom 601 DNA were mixed in a 2.5:1 molar ratio in 10 mM Tris-HCl (pH 7.5) buffer, containing 35 mM NaCl and 1 mM DTT, at room temperature for 20 min. The reconstituted complex was subjected to chromatography on a Superdex 200 10/300 GL gel filtration column (GE Healthcare) in 5 mM HEPES-NaOH (pH 7.5), 35 mM NaCl, and 2 mM TCEP.

### The nucleosome pull-down assay

The nucleosome (1.1 μM) containing the 145 -bp Widom 601 DNA was incubated with GST (2.5 μM), GST-ELYS_C_ (2.5 μM), or GST-ELYS_C_ mutants (2.5 μM of 2R-A, Δ10, R2404A, R2405A, R2406A, R2408A, and R2404A-R2405A-K2406A) at room temperature for 15 min in 20 μL of pull-down buffer (20 mM HEPES-NaOH (pH 7.5), 200 mM NaCl, 0.05% Triton-X, 0.5 mM TCEP, and 0.25 μg/mL BSA). Glutathione Sepharose 4B beads (15 μL) and pull-down buffer (500 μL) were added to the reaction mixtures, which were gently mixed at room temperature for 30 min. The beads were then washed three times with 1 mL of the pull-down buffer without BSA. The proteins that copelleted with the beads were denatured and were analyzed by 18% SDS-PAGE with Coomassie Brilliant Blue staining.

### Preparation of the ELYS_C_–nucleosome for cryo-EM analysis

GST-ELYS_C_ (12 μM) and the nucleosome containing the 145 bp Widom 601 DNA (4.8 μM) were mixed in a 2.5:1 molar ratio at room temperature for 20 min. The GST-ELYS_C_–nucleosome complex was purified and stabilized by the Grafix method^40^. A gradient was formed with buffer A (10 mM HEPES-NaOH (pH 7.5), 35 mM NaCl, 1 mM DTT, and 5% sucrose) and buffer B (buffer A with 20% sucrose and 4% paraformaldehyde), using a Gradient Master (BioComp). The reconstituted GST-ELYS_C_–nucleosome complex (170 μL) was placed on the top of the gradient solution and was ultracentrifuged at 27,000 rpm at 4 °C for 16 h, using an SW32 Ti rotor (Beckman Coulter). One milliliter fractions were collected from the top of the gradient solution by pipetting and were analyzed by 6% non-denaturing PAGE. The collected samples were then desalted with a PD-10 column (GE Healthcare), equilibrated with 10 mM HEPES-NaOH (pH 7.5) buffer containing 2 mM TCEP and were concentrated with an Amicon Ultra centrifugal filter unit (Millipore). The DNA concentration of the sample was 432 μg/mL.

### Cryo-EM specimen preparation and data acquisition

To prepare the cryo-EM specimen, the sample (2.5 μL) was applied to a glow-discharged holey carbon grid (Quantifoil R1.2/1.3 200-mesh Cu). The grids were blotted for 8.0 s under 100% humidity at 4 °C and were plunged into liquid ethane cooled by liquid nitrogen, using a Vitrobot Mark IV (Thermo Fisher). Images were collected using the EPU auto acquisition software on a Talos Arctica cryo-electron microscope (Thermo Fisher), operated at 200 kV, and equipped with a Quantum GIF filter (Gatan) in the energy-filtered transmission electron microscope mode, at a nominal magnification of ×100,000 (pixel size of 1.32 Å). Images were recorded with 10-s exposure times on an energy-filtered (slit width 20 eV) K2 summit direct electron detector (Gatan) in the counting mode, retaining a total of 40 frames with a total dose of ~56 electrons/Å^2^.

### Image processing

In total, 2408 movies of the GST–ELYS_C_–nucleosome complex were aligned and integrated using MOTIONCOR2^41^, with dose weighting. The contrast transfer function (CTF) was estimated by CTFFIND4^42^ from the images, with dose weighting. In total, 1938 images were selected based on the CTF fit correlation to approximately 6 Å resolution. RELION 2.1^43^ and RELION3.0 beta^44^ were used for all subsequent image processing operations. In total, 1,300,821 particles of the GST-ELYS_C_–nucleosome complex were picked automatically with a box size of 160 × 160 pixels. Two rounds of two-dimensional classification to remove bad particles resulted in the selection of 868,748 particles. The ab initio model generated by RELION in the low-pass filter mode to 60 Å was used as the initial three-dimensional model. The class with GST density and the best resolution, containing 285,534 particles, was selected from the first round of three-dimensional classification, and 81,088 particles were further selected based on the CTF fit correlation to approximately 5.5 Å resolution before three-dimensional refinement. The final three-dimensional map was sharpened with an exponential *B*-factor (−218 Å^2^). The resolution of the refined map was estimated by the gold standard Fourier shell correlation (FSC) at the FSC = 0.143 criterion^43^. The final three-dimensional map was normalized with MAPMAN^45^ and was mirrored to match the known handedness of the nucleosome. The local resolution map of the GST-ELYS_C_–nucleosome complex was calculated with RELION^43^. The iso-electron potential surface of the GST-ELYS_C_–nucleosome complex was visualized with UCSF ChimeraX^46^.

### Crosslinking mass spectrometry

ELYS_C_ (11 μM) was mixed with the nucleosome (4.4 μM) containing the 145-bp Widom 601 DNA in a 2.5:1 molar ratio in 10 mM HEPES-NaOH (pH 7.5) buffer, containing 35 mM NaCl and 1 mM DTT, at room temperature for 20 min. After incubation, the sample was crosslinked with 240 μM DSS-H12/D12 (Creative Molecules) at 30 °C for 30 min. The reaction was quenched by the addition of 100 mM ammonium bicarbonate and incubated at 30 °C for 15 min. The crosslinked peptides were prepared for the mass spectrometric analysis^47–50^. The sample was evaporated to dryness and re-dissolved in an 8 M urea solution to a final concentration of 1 mg/mL. The crosslinked proteins were reduced by an incubation with 2.5 mM TCEP for 30 min at 37 °C and were alkylated by a further incubation with 5 mM iodoacetamide for 30 min at room temperature, with protection from light. The sample was diluted with a solution containing 50 mM ammonium bicarbonate to a final concentration of 1 M urea and digested with sequencing-grade endopeptidase Lys-C (Roche) at 37 °C for 2 h (at an enzyme–substrate ratio of 1:50 wt/wt), followed by a second digestion with Trypsin Gold, Mass Spectrometry Grade (Promega), at 37 °C overnight (at a 1:50 ratio wt/wt). The digestion was stopped by the addition of 5% (vol/vol) trifluoroacetic acid (TFA). All peptide samples were purified using C18 spin columns (Pierce) eluted with 50% acetonitrile. The eluted sample was evaporated to dryness and was re-dissolved in water/acetonitrile/TFA, 75:25:0.1. Aliquots (30 μL) of the crosslinked peptides were applied to a Superdex30 Increase 3.2/300 column (GE Healthcare), at a flow rate of 50 μL/min with water/acetonitrile/TFA, 75:25:0.1. Fractions (100 μL) were collected, dried, re-dissolved in 0.1% TFA, and subjected to the liquid chromatography tandem mass spectrometry (LC/MS-MS) analysis. LC/MS-MS analysis was conducted with an LTQ-Orbitrap Velos mass spectrometer (Thermo Fisher Scientific) equipped with a Zaplous Advance nano UHPLC HTS-PAL xt System (AMR). A fully porous particle C18 column (pore size 120 Å, particle size 3 μm, inner diameter 100 μm, length 150 mm) was used for the nano LC, and the gradient, consisting of a linear gradient of the mobile phase developed from 5 to 50% of acetonitrile, was delivered at 300 nL/min over 90 min. The precursor ion was acquired over the mass range of 350–1500 Da, with a resolution of 60,000 full width at half maximum. In the data-dependent scan, single and unassigned charge precursors were rejected, and the ten most intense precursor ions were selected for MS/MS scans. The selected ions were sequentially isolated and fragmented in the linear ion trap by collision-induced dissociation. The crosslinked peptides were identified using xQuest, and the false-discovery rates (FDRs) were estimated by using xProphet^49^. The results were filtered according to the following parameters: FDR < 0.05, minimum *δ* score = 0.85, MS1 tolerance window of –4 –4 ppm, ld-score > 25. The crosslinks were visualized using the webserver xVis^51^.

### Reporting summary

Further information on experimental design is available in the Nature Research Reporting Summary linked to this article.

## Supplementary information

Reporting Summary

Supplementary Information

## Data Availability

The cryo-EM maps of the ELYS_C_–nucleosomes have been deposited in the Electron Microscopy Data Bank, with the EMDB ID code EMD-9802.
