# Supplementary material for: Structural and biochemical analyses of the nuclear pore complex component ELYS identify residues responsible for nucleosome binding
Source: Commun Biol. 2019 May 3;2:163. doi: 10.1038/s42003-019-0385-7 (PMC6499780; doi:10.1038/s42003-019-0385-7)
Supplement: Supplementary file 2 — Supplementary Information [file 42003_2019_385_MOESM2_ESM.pdf]

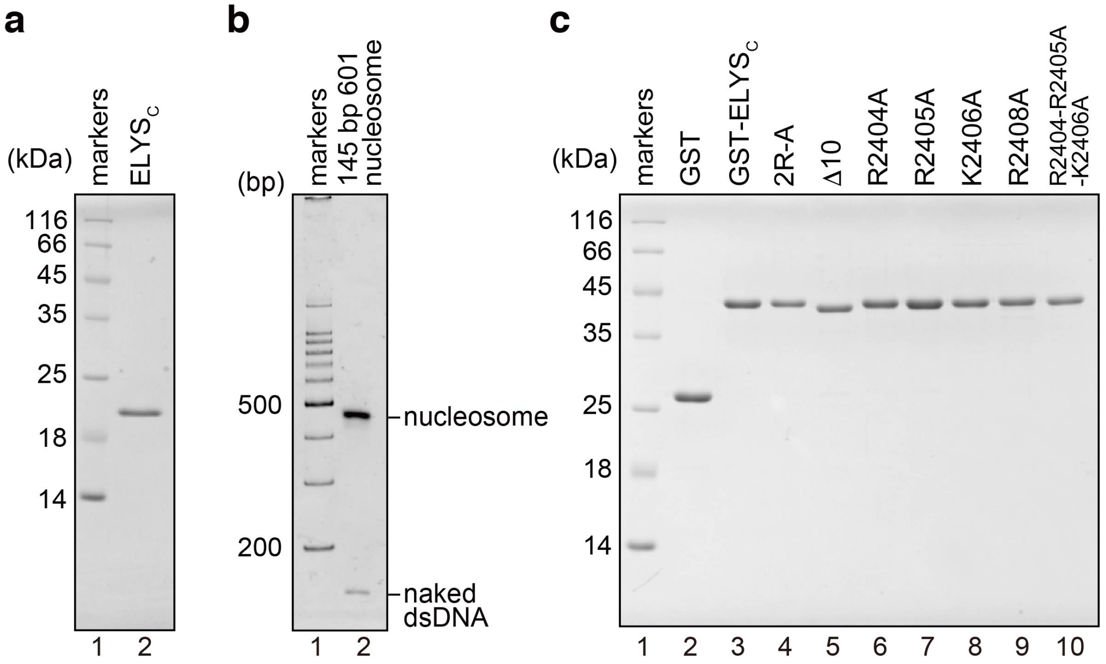

Supplementary Figure 1 Preparation of proteins and nucleosome. **a** Purification of the $ELYS_C$ peptide. Purified $ELYS_C$ (1 μg) was analyzed by 16% SDS-PAGE with Coomassie Brilliant Blue staining. **b** Preparation of the nucleosome containing a 145 bp Widom 601 DNA. The sample was analyzed by 6% non-denaturing polyacrylamide gel electrophoresis with EtBr staining. Lane 1 indicates DNA markers. Lane 2 indicates the purified nucleosome. **c** The GST-fused $ELYS_C$ peptides. Purified GST (0.75 μg), GST-$ELYS_C$, (0.75 μg), and the GST-$ELYS_C$ mutants (0.75 μg) were analyzed by 16% SDS-PAGE with Coomassie Brilliant Blue staining. Lane 1 indicates the molecular mass markers. Lanes 2-10 indicate GST, GST-$ELYS_C$, GST-$ELYS_C$ 2R-A (AT-hook mutant), GST-$ELYS_C$ Δ10, GST-$ELYS_C$(R2404A), GST-$ELYS_C$(R2405A), GST-$ELYS_C$(K2406A), GST-$ELYS_C$(R2408A), and GST-$ELYS_C$(R2404A-R2404A-K2406A), respectively. The uncropped gel images are shown in Supplementary Fig. 4.

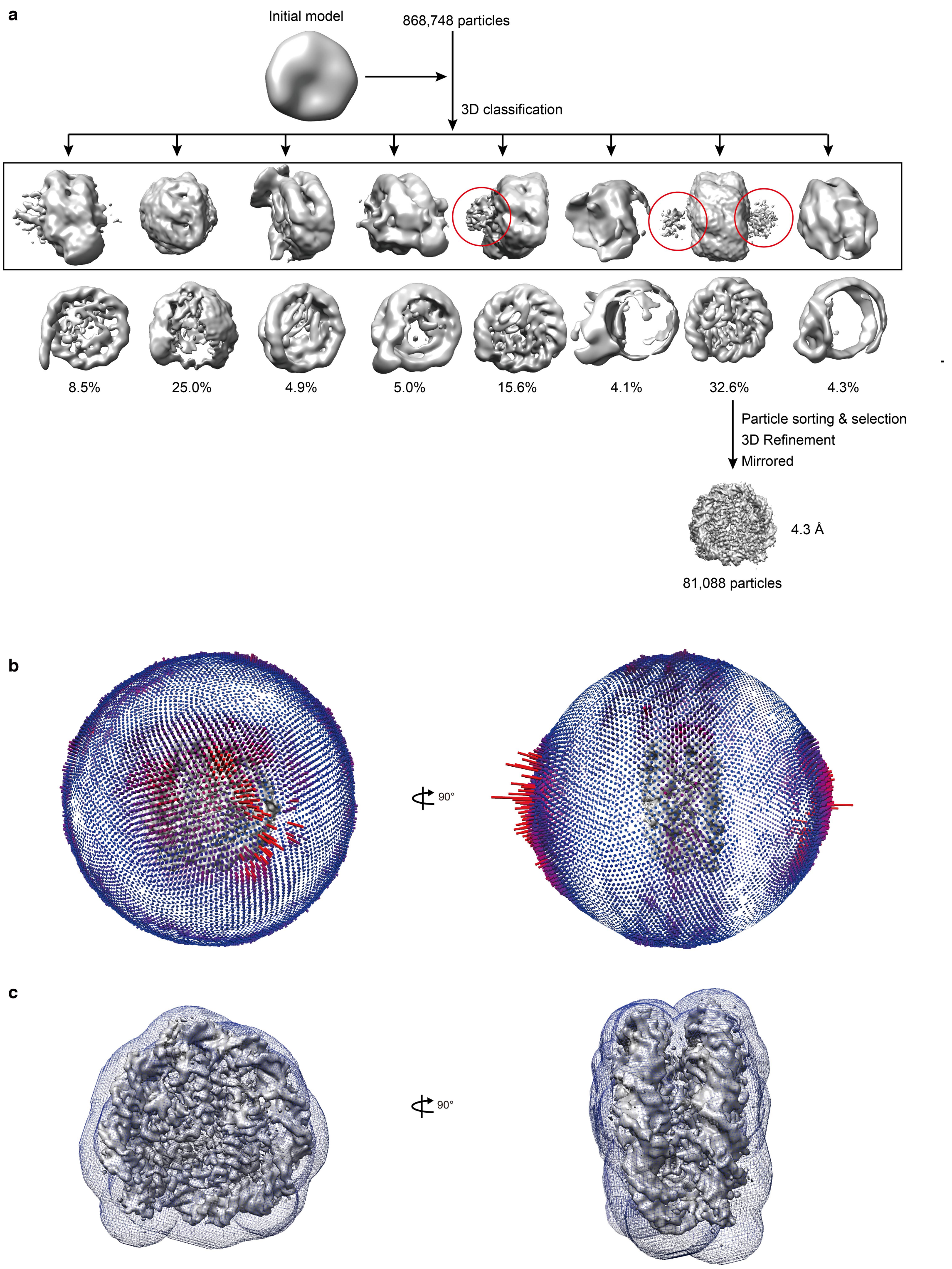

Supplementary Figure 2 Cryo-EM data processing. **a** 3D classification and refinement procedures for the $ELYS_C$-nucleosome complex. In total, 868,748 particles were separated by 3D classification in RELION[43]. The class with GST density and the best

resolution was used to obtain the final cryo-EM density map. Red circles show the GST density. **b** Angular distribution of particle projections of the $ELYS_C$-nucleosome complex. **c** Solvent mask used for 3D refinement.

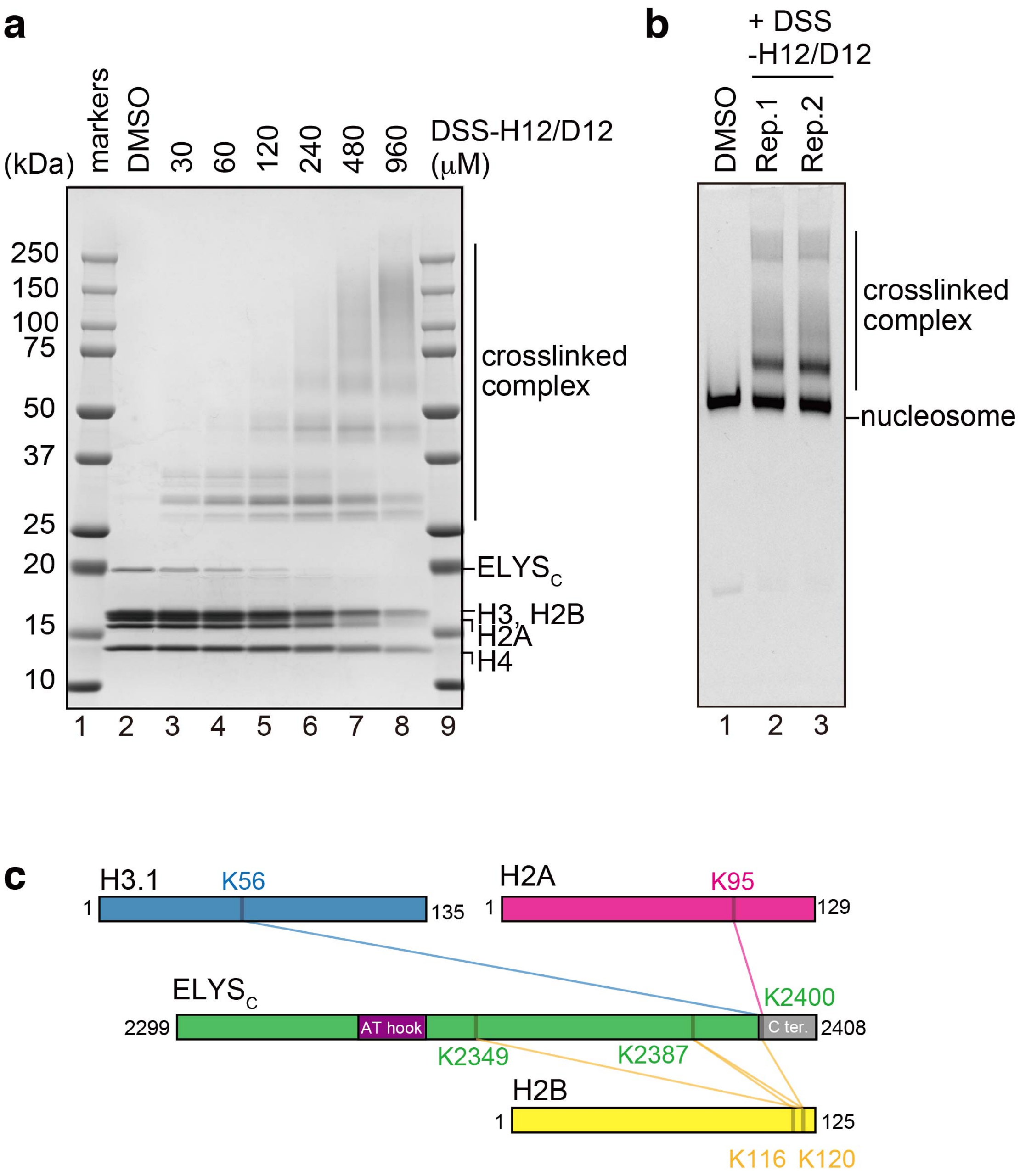

Supplementary Figure 3 Preparation of the ELYS$_C$-nucleosome complex for cross-linking mass spectrometry analysis. **a** ELYS$_C$ and the nucleosome were incubated with the indicated amounts of the DSS-H12/D12 crosslinker (lanes 3-8). Crosslinking efficiency was analyzed by 10-20% SDS-PAGE with Coomassie Brilliant Blue staining. DSS-H12/D12 at 240 μM was chosen for further crosslinking mass spectrometry analysis. Lane 1 indicates molecular mass markers. Lane 2 indicates a control experiment in the presence of DMSO. The uncropped gel image is shown in Supplementary Fig. 4. **b** The crosslinked ELYS$_C$-nucleosome complex was analyzed by

6% non-denaturing polyacrylamide gel electrophoresis with EtBr staining. Lane 1 indicates a control experiment with DMSO. Lanes 2 and 3 indicate two independent replicate experiments. The uncropped gel image is shown in Supplementary Fig. 4. **c** Schematic representation of the replicated crosslinking mass spectrometry analysis. The interlinks between histone core regions (H2A, H2B, and H3.1) and $ELYS_C$ are depicted with lines. The amino acid residues involved in the interlinks are indicated with numbers. The purple and grey boxes represent the regions corresponding to the AT-hook DNA binding domain and the C-terminal 10 amino acid residues, respectively. H3.1, H2A, H2B, and $ELYS_C$ are colored blue, magenta, yellow, and green, respectively.

For Figure 1b

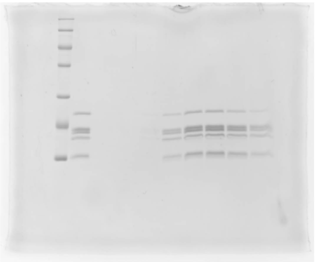

For Figure 1d

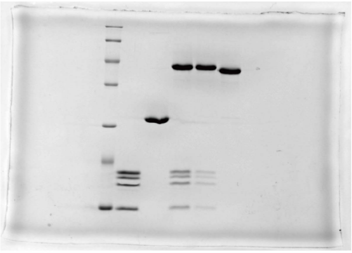

For Figure 2b

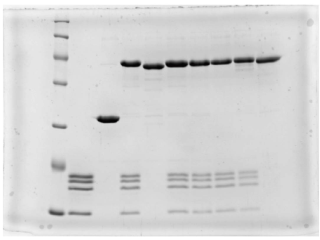

For Supplementary Figure 1a

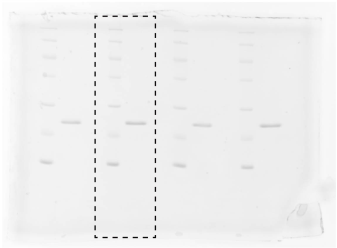

For Supplementary Figure 1b

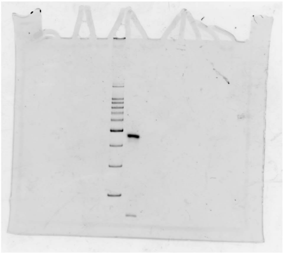

For Supplementary Figure 1c

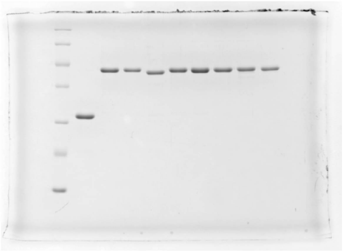

For Supplementary Figure 3a

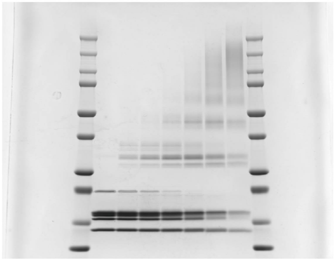

For Supplementary Figure 3b

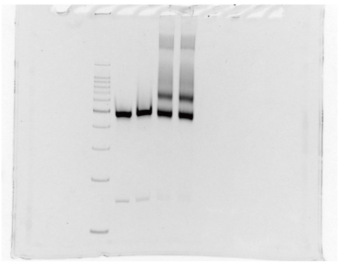

Supplementary Figure 4 Uncropped gel images. The dashed square indicates the region used for Supplementary figure 1a.
